# Serial monogamy benefits both sexes in the biparental convict cichlid

**DOI:** 10.7717/peerj.6535

**Published:** 2019-03-05

**Authors:** Jennifer L. Snekser, Murray Itzkowitz

**Affiliations:** 1 Department of Biology, LIU Post, Brookville, NY, USA; 2 Department of Biological Sciences, Lehigh University, Bethlehem, PA, USA

**Keywords:** Biparental, Cichlids, Reproductive success, Brood success, Retrieval, Parental aggression, Parental care, Sex differences

## Abstract

Monogamy can be either long-term or serial, with new pairs formed with each breeding bout. Costs and benefits are associated with each strategy. Because biparental convict cichlids (*Amatitlania nigrofasciata*) typically switch mates, exhibiting serial monogamy, we tested for the costs associated with forcing individuals to remain with the same mate. Convict cichlids were observed over two successive breeding bouts, either with the same or a new, equally experienced, mate. Parental behavior did not differ between breeding bouts, nor did brood size. Surprisingly, fish that remained with their original partner for a second bout took significantly longer to produce a brood compared to fish that paired with new partners. New partners were also more likely to successfully produce a second brood than re-mated partners. This is in contrast to the majority of bird studies that show many benefits to staying with the same partner for multiple broods. In convict cichlids, there seems to be no benefit associated with remaining with the same partner and switching mates reduces duration between broods for both males and females, potentially increasing overall reproductive success.

## Introduction

Monogamy is observed in a wide variety of animal species, including a limited number of invertebrates, teleost fishes, mammals, and the vast majority of avian species (Clutton-Brock, 1991). With such diversity in the species that exhibit monogamy, it is perhaps unsurprising that there are varying degrees of monogamy, from continuous partnerships with no promiscuity to social monogamy with limited genetic exchange (Black, 1996).

Multiple hypotheses are proposed regarding the costs and benefits associated with long-term mate retention versus serial monogamy (Choudhury, 1995; Black, 1996). Remaining with the same partner may increase familiarity with the mate and/or territory; and increased familiarity and experience with a mate may improve reproductive success. In monogamous birds, brood success is higher in pairs that remain together, though pairing with a new mate for each breeding opportunity (serial monogamy) could be beneficial, if the new mate is of higher quality or maintains better resources (reviewed in birds by Choudhury, 1995, Black, 1996). Of course, procuring a new mate may be costly in terms of time and energy (Sogabe, Matsumoto & Yanagisawa, 2007) and if there are no other individuals available to mate with, then there is little benefit in dissolving an established pairing (Dawkins & Carlisle, 1976). The ultimate expression of the degree of monogamy is likely a product of a complex interaction of species’ life histories, demographics, and/or ecological factors (Kvarnemo, 2018; Lambert, Sabol & Solomon, 2018).

Our aim was to experimentally examine the behavior and reproductive success of monogamous parents when they either remained with the same partner or were given the opportunity to mate with a new partner for a second breeding bout. The majority of studies on monogamy have focused on birds, though a few studies have explored divorce in mammals, such as the alpine marmot (*Marmota marmot*) (Lardy et al., 2011), gibbons (*Hylobatid sp.)* (Palombit, 1994), and the Eurasian beaver (*Castor fiber*) (Mayer et al., 2017). Results from non-avian studies such as these are often in contrast to the patterns we observe in bird species, likely due to differences in behavioral ecology and life-history traits. The convict cichlid fish, *Amatitlania nigrofasciata*, provides an excellent, non-avian, model for understanding monogamy. Convict cichlids have been extensively studied in the field (e.g. Wisenden, 1994, 1995; Wisenden et al., 2008; Snekser & Itzkowitz, 2009; Snekser et al. 2011; van Breukelen, Snekser & Itzkowitz, 2015) and laboratory because of their unique social system, which lends the species to studies on such social behaviors as monogamy, mate choice, biparental care, alloparental care, and aggression.

The convict cichlid is a substrate brooding species native to lakes and streams in Central America, with a distribution including Guatemala, El Salvador, Honduras, Nicaragua, Costa Rica, and Panama (Bussing, 1987). The convict is a relatively small cichlid; breeding individuals are between 40 and 80 mm standard length (SL) (Wisenden, 1995). In Costa Rican streams, convict cichlids breed throughout the long dry season from December until June and may survive for two breeding seasons (Wisenden, 1994). Adults form monogamous pairs and both parents defend the nest and young for six to eight weeks. Females lay approximately 200 eggs on a hard surface during a single breeding bout. During this time, both parents are seen in or near the nest. After approximately 3 days, the eggs hatch and the yolk-sac larvae (“wrigglers”) that emerge from the eggs are unable to swim for an additional 4–5 days and continue to be closely guarded by the parents. Following absorption of the yolk, the young become free-swimming and begin to feed by foraging within the substrate. The young remain in a shoal near their parents and potential predators are driven away by the parents.

While both parents contribute to parental care, each parent has a specific role. Males engage mostly in territorial defense and protecting young from predation, whereas females remain near the offspring (Wisenden, 1995; Snekser & Itzkowitz, 2009, 2014). These sex-specific behaviors are emphasized in the presence of a mate or an intruder in laboratory studies (Itzkowitz, Santangelo & Richter, 2001). These activities, however, are not obligate: parents do switch roles for brief periods of time and, in the absence of one parent, each sex can perform all necessary parental activities (Itzkowitz, Santangelo & Richter, 2001, 2002). Additionally, females defend against a conspecific intruder just as much as male partners when the female is larger than the male parent and the intruder is of equivalent size (Itzkowitz et al., 2005).

In a multi-year study in Costa Rica, only 1 of 59 convict cichlid fish that produced a second brood (within the same year or the following breeding season) did so with the same mate (Wisenden, 1995). It has been hypothesized that this serial monogamy occurs because males can mate multiple times but females generally only produce one brood each breeding season (Wisenden, 1995). Because of this sexual asymmetry, a male should switch mates, thus eliminating any time that he would have to wait for his initial mate to replace her ova (Baylis, 1981). However, 72% of males did not re-mate within a breeding season (Wisenden, 1995) suggesting that either receptive females were unavailable or other factors were responsible for not re-mating with the same female. This behavior is different than that observed in many avian species in which mate fidelity increases reproductive success (e.g. Hatch & Westneat, 2007), strengthens coordination between pairs (e.g. Choudhury, 1995; Ihle, Kempenaers & Forstmeier, 2015), and also decreases time between broods (e.g. Lifjeld & Slagsvold, 1988).

Here, we allowed convict cichlids to immediately mate for a second time, either with the same mate or a new mate. Based on the behavior previously observed in natural populations, that convict cichlid pairs rarely remain monogamous naturally, we examined if re-pairing affected parental behavior. If one or both members of a pair preferred not to mate with the same partner, we assessed whether this possible reluctance would carry over into their parental care behavior. More specifically, we predicted that pairs that remain together would show less precise coordination of their parental division of roles than they did during their first mating or when compared to newly paired mates. Less coordination would mean that the male and female would spend less time performing their sex-specific roles (Itzkowitz et al., 2005; Snekser & Itzkowitz, 2009). We also examined the brood size and the time to spawn for pairs that re-mated or mated with new partners. If there is a preference to mate with new partners, we predicted that more young would be produced by the pairs with new partners, as compared to pairs that remained with the same partner. If time to produce new offspring is based solely on the female’s ability to produce new mature ova, we predicted that all pairs should take equally long to spawn, given that all fish had just produced a brood. If timing to produce a new brood is related to willingness or eagerness to mate with a specific partner, we would predict that, for convict cichlids, partners with new pairs would produce broods more quickly than partners who remained with their previous mate.

## Materials and methods

Experiments were conducted in accordance with institutional guidelines under Lehigh University’s IACUC (Animal Welfare Assurance No. A3877). Convict cichlids were from laboratory stock populations derived from pet store and wild-caught fish. Fish were kept on a 12:12 L:D photoperiod at 20 ± 2 °C and fed pellet food each day. One male and one female convict cichlid were placed in a 284-L test aquarium. Pairs were arranged based on size, with male fish 10 mm SL longer than females, as is typical in natural populations (Wisenden, 1995). Fish used within this experiment had no known breeding experience.

Each test aquarium contained a clay pot for nesting. At the opposite end of the tank, a 15 cm “intruder area” was partitioned using clear plexiglass and a small (<30 mm SL) cichlid was placed in this area to increase pair bonding (Itzkowitz & Draud, 1992). This small intruder was removed following the formation of a pair bond and egg laying. For this first breeding bout, if the pair did not spawn within one month, the replicate was ended.

Eggs were laid within the flower pot and within 3 days hatched into non-mobile larvae, called wrigglers. Testing began within 24 h of hatching. In order to test indirect parental care, a male intruder (10 mm SL larger than the male of the pair) was added to the intruder area five minutes prior to and removed following testing. After the intruder was added, all wrigglers were removed from the nest using a 10 mL pipette and counted. In order to test direct parental care, 50 wrigglers were placed in the center of the tank, equidistant between the nest and the intruder area. These displaced wrigglers are unable to swim and parents will return them to the nest (Snekser & Itzkowitz, 2009, 2014). Broods were typically larger than 50 young, and remaining wrigglers were returned to the nest.

Behavior was video recorded for one hour. Behaviors were scored using JWatcher (UCLA & Macquarie University) from the time of placement of wrigglers until the last wriggler was retrieved. This final retrieval was determined by visually confirming that all wrigglers were retrieved when recording ended and then watching the entire video. Only the behavior exhibited by parents during the retrieval period was analyzed, because time spent retrieving varied among pairs. Four parental behaviors were recorded: number of wriggler retrievals (number of times a parent put their snout down to the immobile displaced wrigglers and picked them up with their mouths), rate of aggression toward intruder (number of bites or charges per minute), and the percentage of time spent within one body length of the intruder and within the nest (pot). The proportion of time spent together at the intruder and that parents spent with strict role division (i.e., female near nest, male near intruder) were calculated. Due to the variability in the total amount of time that behaviors were observed, the relative proportion of time spent engaged in each activity and the rate of aggressive behavior were used within analyses. The exact number of retrievals of displaced young performed by each parent were used for analysis, as each replicate had the same number (50) of wrigglers displaced/retrieved.

In order to determine if behaviors or reproductive success would differ between pairs that remained together and pairs that re-spawned with new (equally experienced) partners, all fish were placed into new test tank immediately following the second day of testing. All previously paired fish were placed into a new tank with opaque black plexiglass dividing it in half. The breeding pair was visually separated, with one adult placed on either side of the black partition. After 48 h, the black partition was lifted and a clay pot and clear intruder area partition were placed in a set-up identical to the first breeding bout. For this second bout, two groups were examined: those that remained with the same partner (*N* = 9) and those paired with a new partner (*N* = 9). Pairs with new partners were comprised of two individuals that had previously spawned and had their wrigglers displaced from the nest, and whose behavior was recorded during the initial breeding bout. Therefore, all parents in all pairs had identical experiences with spawning and parenting and only differed in the fish with whom they were paired (same or new partner). Pairs were given up to 6 weeks to spawn. When all eggs of the second breeding bout hatched, behavioral testing occurred exactly as during the initial breeding bout (described above).

All statistics were performed with SPSS and normality of data was confirmed with the Kolmogorov-Smirnov test. Success of second brood production for replicates was compared using a Pearson Chi-square. Brood sizes and days to spawn between the two breeding bouts were compared separately for males and females and for pairing (same or different partner) using Paired-sample t-tests. To test the hypothesis that pairing status affects reproductive success, a two-factor mixed-design ANOVA (General Linear Model (GLM)) was used to compare males and females (within subjects factor) and same/new pairing (between subjects factor) for the difference in eggs produced (brood size of second bout—brood size of first bout) and the difference in days to spawn (days to spawn second bout—days to spawn first bout). To test the hypothesis that parental behavior will differ between pairs and that male and female parents behave differently, the four parental behaviors recorded (proportion of time spent in the nest with non-displaced offspring, number of retrievals, proportion of time spent near the intruder, and the rate of aggression), the proportion of time that parents spent together at the intruder, the proportion of time that parents performed their sex-typical roles, and the total time spent collecting wrigglers were analyzed in separate three-way factorial Repeated Measures ANOVAs (GLM) (within subjects factors: sex (male/female); breeding bout (first/second); between subjects factor: partnering (same/new)).

## Results

A second breeding bout was attempted for 34 pairs and was successfully produced by nine pairs that remained together and nine pairs in which the mates were new partners. Sixteen pairs failed to spawn a second time. The failed replicates were due to aggression between partners (sometimes initiated by the male, sometimes initiated by the female), consumption of laid eggs (which may or may not have been fertilized), or failure to breed again within 6 weeks. Of the failed replicates, 11 were pairs that remained together and five were pairs of new mates. Failure rates were not statistically different (χ^2^ = 1.2295, *p* = 0.2675).

**Figure 1:**
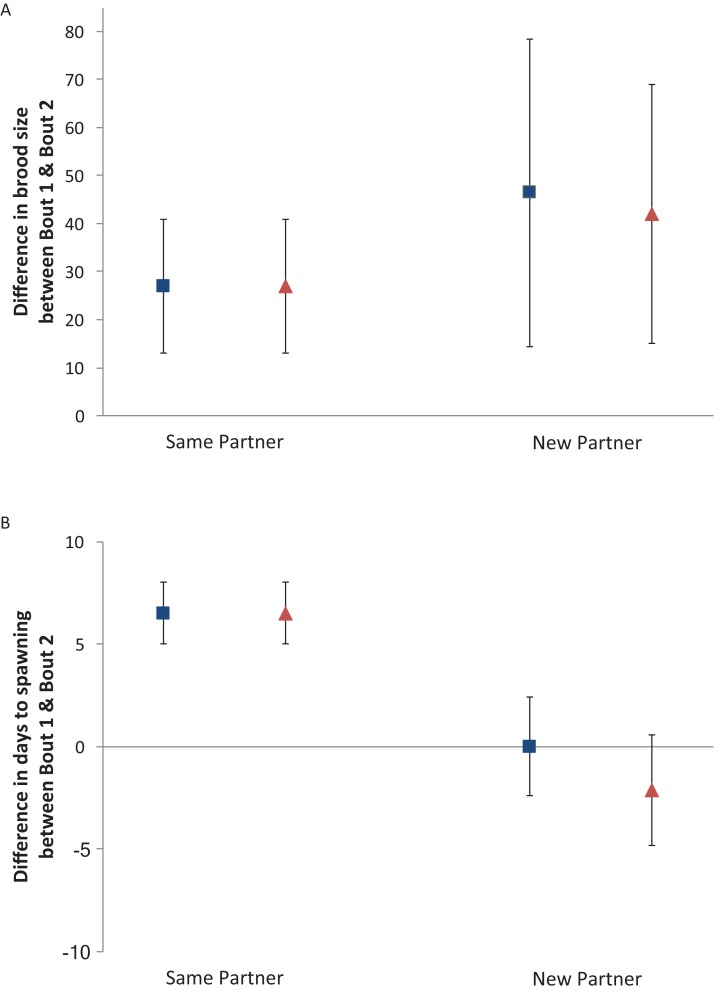
Difference in brood size and days until spawning between first and second breeding bout. (A) Mean ± SE difference between breeding bouts in brood size and (B) Mean ± SE difference between breeding bouts in days to spawning for male (blue) and female (red) convict cichlids that remained together or mated with new partners.

In pairs that remained together, brood sizes were not statistically different between breeding bouts (df = 8, *t* = −1.197, *p* = 0.084) with a mean ± SE brood size during the first breeding bout of 132 ± 10.69 eggs and 159 ± 12.20 eggs during the second bout. In pairs that mated with new partners, brood sizes did not differ for males (df = 8, *t* = −1.456, *p* = 0.183) or females (df = 8, *t* = −1.580, *p* = 0.153) with a mean ± SE brood size of 137 ± 14.07 eggs for males and 142 ± 14.37 eggs for females in the first bout and 184 ± 24.07 eggs in the second. Comparison of the difference in brood size (wrigglers in first breeding bout—wrigglers in second breeding bout) revealed no differences between the sexes (*F* = 0.034, *p* = 0.826), no differences between parents paired with either the same or new partners (*F* = 0.215, *p* = 0.649), nor any interaction between sex*partnering (*F* = 0.034, *p* = 0.826) (Fig. 1A).

Pairs that remained together for a second bout took significantly longer to spawn the second time compared to the first (df = 8, *t* = −4.341, *p* = 0.005) with a mean ± SE of 10.88 ± 2.96 days before the first and 17.44 ± 3.94 days before the second bout. When pairs were comprised of individuals that had not mated together before, neither males nor females exhibited differences in days to spawning (males: df = 8, *t* = 0.000, *p* = 1.000; females: df = 8, *t* = 0.775, *p* = 0.461). Males took a mean ± SE of 15.34 ± 2.03 days to spawn before the first bout and females took 17.44 ± 2.44 days. For the second bout, it took an average ± SE of 15.34 ± 1.86 for pairs to spawn. Comparison of the difference in spawning rates (days to spawn in first breeding bout—days to spawn in second breeding bout) indicate that pairs that remained together took significantly longer to produce a second brood than parents that were re-paired with new mates (*F* = 5.205, *p* = 0.006). There was not a significant effect of parental sex (*F* = 0.358, *p* = 0.558), nor any significant interaction of sex*partnering (*F* = 0.358, *p* = 0.558) (Fig. 1B).

**Figure 2:**
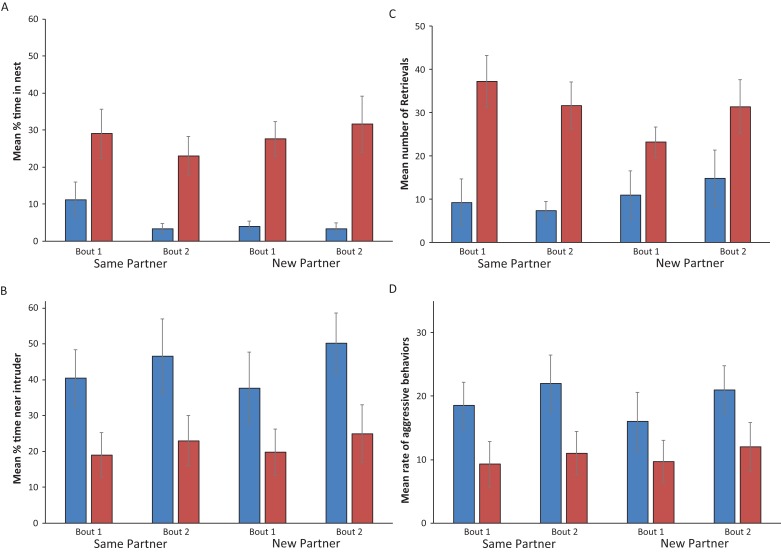
Direct and Indirect parental behaviors exhibited by convict cichlids with the same or new partners across two breeding bouts. Mean +/− SEM (A) proportion of time spent in the nest (B) number of wriggler retrievals (C) proportion of time spent engaged in aggression with an intruder and (D) rate of aggression by male (blue) and female (red) parents with the same or new partners across two breeding bouts.

A significant effect of sex was apparent for the indirect and direct parental behaviors exhibited. Females spent more time within the nest and retrieved more wrigglers. Males spent significantly more time near a conspecific intruder and had a significantly higher rate of aggression. There were no significant effects of breeding bout (first or second) or partnering (same or new partner), nor any interaction (Fig. 2; Table 1). The only exception to this is a significant interaction of bout*partnering for the number of retrievals, which appears to be due to fewer overall retrieval behaviors displayed by newly partnered parents during their second bout (though it should be noted that all 50 wrigglers were retrieved by these pairs, as they were for all pairs) (Fig. 2; Table 1). Additionally, there was no effect of partnering nor breeding attempt, nor any interaction of the two on the proportion of time that parents spent together at the intruder nor on the proportion of time that parents performed their sex-typical roles (Table 2). Total wriggler collection times ranged from 136 s to 2,206 s. Pairs that remained together, on average, took 52 s longer during the second breeding bout, compared to the first, to collect their 50 displaced offspring. Parents with different partners retrieved their offspring 265 s faster, on average, during the second breeding bout compared to the first. These differences were not statistically significant (Table 2).

**Table 1:** Statistical analyses of direct and indirect parental behaviors.

Behavior	Effect	*F*	*p*
% Time in nest	Sex	28.643	**<0.001**
	Sex*Partnering	0.778	0.391
	Sex*Bout	0.511	0.485
	Bout	0.979	0.337
	Bout*Partnering	2.499	0.133
	Bout*Partnering*Sex	0.121	0.733
	Partnering	0.000	0.991
Retrievals	Sex	18.574	**0.001**
	Sex*Partnering	1.224	0.285
	Sex*Bout	0.003	0.955
	Bout	0.181	0.677
	Bout*Partnering	7.628	**0.014**
	Bout*Partnering*Sex	0.098	0.759
	Partnering	0.086	0.773
% Time near intruder	Sex	22.563	**<0.001**
	Sex*Partnering	0.016	0.900
	Sex*Bout	0.264	0.614
	Bout	1.876	0.190
	Bout*Partnering	0.141	0.712
	Bout*Partnering*Sex	0.076	0.786
	Partnering	0.015	0.904
Rate of aggression	Sex	18.599	**0.001**
	Sex*Partnering	0.321	0.584
	Sex*Bout	0.346	0.565
	Bout	1.800	0.198
	Bout*Partnering	0.057	0.814
	Bout*Partnering*Sex	0.010	0.912
	Partnering	0.012	0.913

**Notes:**

Statistical results of three-way factorial Repeated Measures ANOVAs (GLM) (within subjects factors: sex (male/female); breeding bout (first/second); between subjects factor: partnering (same/new)).

Significant *p*-values are given in bold.

**Table 2:** Statistical analyses of total collection time and behaviors associated with coordination.

Behavior	Effect	*F*	*p*
% Time parents together at intruder	Bout	0.927	0.345
	Bout*Group	0.000	1.000
	Group	0.022	0.978
% Time male at intruder and female at nest	Bout	1.046	0.317
	Bout*Group	0.119	0.888
	Group	0.338	0.716
Total time spent collecting offspring	Bout	1.087	0.307
	Bout*Group	0.481	0.624
	Group	0.821	0.452

**Note:**

Statistical results of three-way factorial repeated measures ANOVAs (GLM) (within subjects factors: sex (male/female); breeding bout (first/second); between subjects factor: partnering (same/new)).

## Discussion

Convict cichlids exhibit serial monogamy, rarely re-mating with the same mate for a second breeding bout (Wisenden, 1995), leading to the hypothesis that pairs of convict cichlids that remain together after an initial mating incur some sort of cost. Our experimental data support this hypothesis: pairs that remained together took longer to produce the next brood than those that switched mates. They also took longer to lay eggs for the second brood compared to the first. Pairs that remained together took almost a week longer, on average, to lay a second clutch of eggs compared to fish that spawned with new mates. Considered over the entire multi-month breeding season and throughout the lifetime of a convict cichlid (which can include multiple breeding seasons), this acceleration in brood production when switching partners could lead to a significant effect on the overall reproductive success of those fish that readily switch partners.

We reject the previous hypothesis that the delay in brood production is caused by the time it takes females to mature her next batch of ova (Baylis, 1981; Wisenden, 1995). Both males and females provided with a new, equally experienced, mate spawned significantly faster than those fish forced to maintain monogamy with the same partner. If the time it took to spawn for a second time were based on the maturation of eggs alone, we would have expected females in both groups to spawn in a similar amount of time.

We also found that mate fidelity had no effect on the parental behaviors we examined. In every pair, males spent significantly more time engaged in aggressive behavior and exhibited a higher rate of aggression while females retrieved significantly more of the displaced young and spent significantly larger proportion of time in the nest, as seen in previous studies on convict cichlids examining these direct and indirect parental behaviors (Snekser & Itzkowitz, 2009, 2014). There was also a significant interaction of paring and breeding bout in terms of the number of retrievals, which appears to be due to more retrievals being performed during the first breeding bout by pairs that eventually remained together, compared to pairs that eventually paired with new partners, though we suspect that this may be an artifact of small sample size. In a further exploration of parental behaviors, no difference was apparent for the total time spent collecting displaced young or either measure of behavioral coordination (dividing sex-typical roles or both parents engaging in aggression with the intruder simultaneously). Thus, once the re-mated pair produced the next batch of offspring, their parental behavior was not different from their initial mating nor from pairs with a new mate.

The general lack of benefit of mate fidelity sheds some light on the serial monogamy naturally displayed by these fish in natural populations (Wisenden, 1995). Behaviorally, we found no difference in parental behavior exhibited if a fish maintained a pairbond or formed a new one. Spawning with a new partner resulted in fewer days between bouts and slightly larger broods. With the lack of benefit of fidelity, pairing with new partners each breeding season may lead to individual benefits from the possibility of exchanging genes with a better mate (Ens, Safriel & Harris, 1993). Convict cichlids do not appear to be making a new mate choice prior to divorce (Wisenden, 1995), so assessment to determine if the new mate is better than the previous does not appear to be playing a role in the decision to depart from a previous partner. Without the adaptive benefits associated with multi-year pair bonds, there is no profit in perennial monogamy.

It is interesting to consider the stark differences seen when comparing this study on biparental fish with previous studies on avian parents. Often, birds that remain with the same partner have greater reproductive success than those that experience divorce (Bradley et al., 1990; Dubois & Cézilly, 2002; Adkins-Regan & Tomaszycki, 2007; Crino et al., 2017). For example, zebra finch (*Taeniopygia guttata*) pairs that remained together laid a second clutch significantly faster than divorced parents (Adkins-Regan & Tomaszycki, 2007; Crino et al., 2017) and their clutch mass was significantly higher (Crino et al., 2017). Interestingly, parental behaviors (incubation and feeding rate) were not significantly different between the two groups of divorced and faithful zebra finch parents (Crino et al., 2017), similar to our results in convict cichlids, but in contrast to previous studies of other birds in which mate fidelity was shown to significantly increase the behavioral coordination within pairs (e.g. Choudhury, 1995; Ihle, Kempenaers & Forstmeier, 2015).

It seems that the most parsimonious explanation for these taxa differences is related to differences in ecological demands of parental care and the resultant differences in parental strategies. Typically, bird parents must provide their altricial nestlings with copious amounts of food and also remain vigilant for predators. Because of these two demanding and conflicting activities, each parent takes on each necessary activity (foraging for food for their young and guarding nestlings) for approximately fifty percent of the time, displaying what is often referred to as a “division of labor” (e.g. Wright & Cuthill, 1989, 1990; Schwagmeyer, Mock & Parker, 2002; Schwagmeyer, Schwabl & Mock, 2005; Harding et al., 2004). In contrast, cichlid parents mainly provide care to the young by protecting them from predators. While swimming with parents, the small shoal of fry feed upon the detritus, with parents occasionally digging or lifting leaves to circulate more detritus for them. With these demands, cichlid parents typically take a strategy of males performing the majority of defensive behaviors and females directly interacting with young, often termed a “division of roles” (Smith-Grayton & Keenleyside, 1978; Itzkowitz, 1984; Keenleyside, 1985; Wisenden, 1995). Because these biparental fishes have different ecological demands and therefore a different approach to parental care than avian parents, there may not be a benefit to the coordination that is assumed to result from pairing with the same mate for subsequent reproduction. For the cichlids, parental roles are sex-typical and generally seem to be well-defined within each pair. This is evidenced in our findings that parental behaviors did not change from one breeding bout to the next, nor did behavior differ between parents that divorced or remained with the same partner. The inherent sex differences already result in coordination of parental behavior. Familiarity of a partner may not increase this behavioral coordination substantially, and therefore the greater benefit lies in reproducing with another partner (i.e., genetic diversity in offspring).

Our results support the hypothesis that, for parental animals that exhibit a strict “division of roles” with sex-typical coordination of behavior, there is little reproductive benefit in remaining with a partner because mate-familiarity does not appear to improve parental care nor overall reproductive success. In fact, divorced pairs in which parents had not previously mated with each other laid eggs significantly faster than pairs that remained monogamous. Though not significantly different, they were also twice as likely to spawn for a second time. This finding is somewhat in agreeance with findings from mammalian species in which monogamous pairs are dissolved and parents re-mate with new partners and overall reproductive success (as measured by number of young) did not change with new partnerships (Lardy et al., 2011; Mayer et al., 2017). Mammalian parental care much more closely resembles a “division of roles” (with females providing milk and males contributing in other ways, if at all) than it does a “division of labor”. This congruence is in line with our hypothesis that the benefits of mate fidelity will be linked to the parental tactics of a species. For the convict cichlids, there appears to be a benefit to pairing with a new mate, at least in terms of timing of brood production.

We do not yet understand the mechanism by which the difference in the time to spawning occurs. It is possible that pairing with the same mate caused stress and indecisiveness within the pair, thereby leading to longer spawning rates. Perhaps the first breeding bout was perceived to be a failure due to our removal of young before completion of the typical parental care period, and thus neither parent was eager to engage in another pair bond with their previous partner. Female convict cichlids do seem to be able to adjust the number of eggs laid in relation to external factors (Wisenden, 1993) and they may be adjusting eggs laid depending on some characteristics (such as familiarity) of their mate. Both male and female convict cichlids assess mates as part of the pair-bonding process (Leese, 2012). It is unclear from this study if the male or the female in the pair is initiating the divorce, but it does seem that both sexes are benefitting from finding a new mate.

## Conclusions

The aim of this laboratory study was to experimentally examine the behavior and reproductive success of monogamous parents when they were forced to remain with the same partner or were given the opportunity to mate with a new partner (of equal reproductive experience) for a second breeding bout. In the field, convict cichlids typically exhibit serial monogamy, finding new mates with each breeding bout. Reproductive benefits, such as reduction in time to initiating clutch and increased clutch size are typically associated with avian parents who remain with the same partner. Our results indicate that no such benefit is associated with strict monogamy in the convict cichlid. Direct and indirect parental behaviors and number of eggs laid did not differ between pairs that remained with their partners and those that reproduced with new partners. New partners laid their second brood significantly faster than pairs that remained together and, compared to pairs of new partners, more than twice as many monogamous pairs failed to reproduce a second time. These results are in contrast to avian biparental care studies and suggest that both male and female convict cichlids benefit from serial monogamy. Further investigation is necessary to better understand which parent is initiating divorce, at what stage of fry development this occurs, if mate searching occurs following divorce, and how choice influences reproductive success, as well as the proximate mechanisms that influence these aspects of parental care.

## Supplemental Information

10.7717/peerj.6535/supp-1 Supplemental Information 1 Dataset for Snekser & Itzkowitz serial monogamy experiment. All data used in analysis for all males and females during their two breeding bouts are included. Behaviors, egg counts, and days to spawning are all included.

## Additional Information and Declarations

### Data Availability

The following information was supplied regarding data availability: The raw data is included in Supplementary File 1.
